# Posttranslational modifications of phosphodiesterase type 4 enzymes represent novel points for therapeutic targeting

**DOI:** 10.1111/febs.70205

**Published:** 2025-07-27

**Authors:** Madihah Hussain, Gonzalo S. Tejeda, George S. Baillie

**Affiliations:** ^1^ School of Cardiovascular and Metabolic Health, College of Medical Veterinary and Life Sciences University of Glasgow UK

**Keywords:** cyclic AMP, phosphodiesterase type 4, posttranslational modification

## Abstract

Cyclic AMP is a second messenger that is produced in response to the activation of many G‐protein‐coupled receptors. As each receptor type is linked to a transient but distinct physiological outcome, the activation of cAMP effector proteins is highly compartmentalized by the action of phosphodiesterases (PDEs). Phosphodiesterase type 4 (PDE4) enzymes are expressed as 25 different isoforms, and the function of each protein is linked to its cellular location(s). Fine‐tuning of cAMP dynamics in space and time is underpinned by PDE4 activity shifts or PDE4 translocations that are driven by posttranslational modifications. As ‘omics’ technology improves, we are now learning more about these PDE4 events, and we can link them to diseases where aberrant cAMP signaling is causative. Additionally, recent advances allow us to pinpoint specific PDE4 modifications with targeted therapies that will lessen the chances of side effects. This review charts all known PDE4 modifications and links them to innovative existing pharmaceutical concepts or possible future therapeutic developments.

## Graphical abstract

Enzymes from the phosphodiesterase type 4 family (PDE4) coordinate physiological events driven by the cyclic AMP s(cAMP) signaling pathway by tightly controlling cAMP dynamics in many cell types. Key to this strict regulation is the myriad of posttranslational modifications that alter PDE4 activity, cellular location, and binding partner selection. This review explains how different PDE4 modifications specifically alter PDE4 function and how these alterations can be targeted with novel pharmacologies.

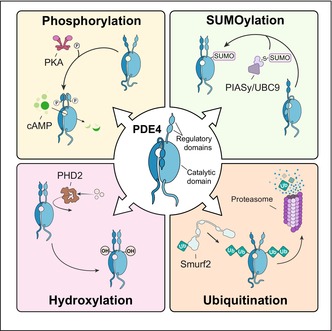

**Abbreviations**
AMPK: AMP‐activated protein kinase
CaMKII: calcium/calmodulin‐dependent protein kinase II
cAMP: cyclic AMP
CK1: casein kinase 1
Cdk5: cyclin‐dependent kinase 5
E1: ubiquitin‐activating enzymes
E2: ubiquitin‐conjugating enzymes
E3: ubiquitin‐protein ligases
GSK3β: glycogen synthase kinase 3β
MK2: MAPK‐activated protein kinase 2
PDE: phosphodiesterase
PHD2: prolyl hydroxylase enzyme
PHICS: phosphorylation inducing chimeric small molecules
PKA: protein kinase A
PROTAC: Proteolysis‐Targeting Chimera
PTM: posttranslational modification
SIK1: salt‐inducible kinase 1
SUMO: small ubiquitin‐like modifier
UCR1: upstream conserved region 1

## Introduction to Phosphodiesterases

Phosphodiesterases (PDEs) are the only group of enzymes capable of hydrolyzing the cyclic nucleotides that act as second messengers [1]. These intracellular emissaries function by activating cell signaling cascades to alter cell physiology in response to extracellular stimuli that triggered their production. As the range of cellular processes influenced by these second messengers is varied, a complex array of PDEs is required to coordinate cyclic nucleotide dynamics in the cytoplasm. To this end, cells express a superfamily of over 100 isoforms that are encoded by 11 families (PDEs 1–11). Each isoform has a differential affinity for cyclic nucleotides [cyclic AMP (cAMP) or cyclic GMP (cGMP)], distinct cellular location, and unique abilities to be regulated by posttranslational modifications (PTMs) of different kinds [2]. Reversible PTM allows fine temporal and spatial control over a PDE enzyme's activity, and this is key to shaping cyclic nucleotide gradients. These gradients are tailored to breach the threshold of activation of a unique subset of different cyclic nucleotide effector proteins for each cell surface receptor‐mediated event [3].

The sequences of the different PDE families can be divided generally into three types of domains: catalytic, targeting, and regulatory [4]. Although all the catalytic domains are structurally related and perform the same function, that is, hydrolysis of cyclic nucleotides, they are different enough to be targeted by family‐specific small molecule inhibitors. As the cellular location of a PDE isoform is correlated with its function, unique ‘postcode’ sequences on the protein's surface often target the enzyme to precise organelles, cellular structures, or signaling complexes to influence cyclic nucleotide dynamics in distinct intracellular compartments. Arguably, the greatest divergence between PDE family members is the manner in which they get regulated by PTMs. This provides another level of functionality for the PDE superfamily and promotes crosstalk with signaling cascades other than those involving cyclic nucleotides.

**Fig. 1:**
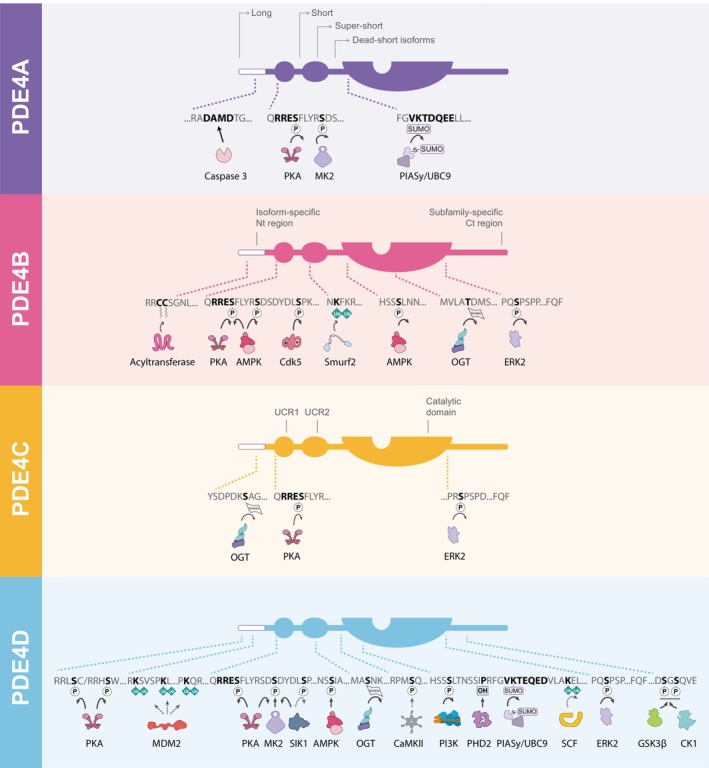
Posttranslational modifications (PTMs) of PDE4 subfamilies. PDE4 domain structure and PTM sites are shown together with the regulating enzymes responsible for the phosphorylation, proteolysis, ubiquitination, SUMOylation, hydroxylation, *O*‐GlcNAcylation, and acylation of each subfamily. Dashed lines indicate the location of the PTM site sequence in the schematic PDE4 structure. Modified amino acids are highlighted in bold. Dashed arrows denote PTMs that require further confirmation. Sequences separated by slash indicate isoform differences in the amino acid sequence (PDE4D7/PDE4D3). AMPK, AMP‐activated protein kinase; CaMKII, calcium/calmodulin‐dependent protein kinase II; Cdk5, cyclin‐dependent kinase 5; CK1, casein kinase 1; ERK2, extracellular signal‐regulated kinase 2; GSK3β, glycogen synthase kinase‐3β; MDM2, mouse double minute 2; MK2, MAP kinase‐activated protein kinase 2; OGT, O‐GlcNAc transferase; PHD, prolyl hydroxylase domain; PI3K, phosphoinositide 3‐kinase; PIASy, protein inhibitor of activated STAT y; PKA, protein kinase A; SCF, skp1, cullin, F‐box containing complex; SIK1, salt‐inducible kinase 1.

Of the 11 different families, the most comprehensive literature set is available for the PDE4 family. Although this family has the most complex expression pattern, with at least 25 different isoforms being encoded by four genes, there are readily available and specific antibodies, biotools, and knockout mice for the subfamilies (PDE4 A, B, C, D) [5]. Interest is also peaked by PDE4 involvement in a range of diseases and the availability of clinically approved inhibitors, with a range of further PDE4‐specific compounds in clinical trials [6]. This review will cover all the reported PTMs for PDE4 enzymes and will consider their relevance to the therapeutic targeting of this family. A diagrammatic overview of the PTMs covered here is provided in Fig. 1.

## PDE4 structure

**Fig. 2:**
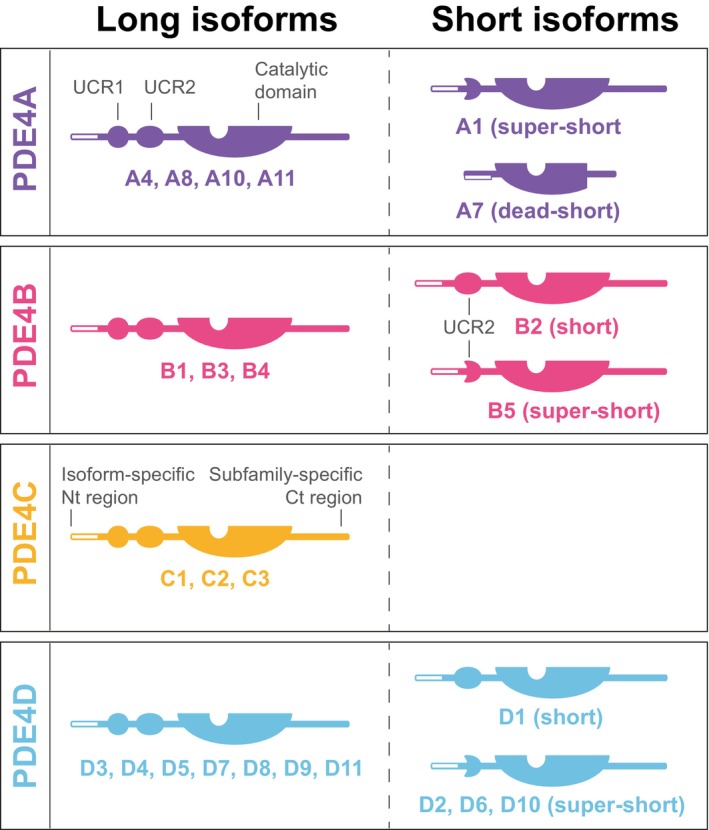
PDE4 modular structure. PDE4 subfamilies are encoded by four genes, PDE4 A (purple), B (pink), C (orange) and D (blue). Each isoform is characterized by the expression of different domains that are regulated by alternate mRNA splicing and promoter diversity. Each subfamily has a unique conserved C‐terminal domain and catalytic domain, and each isoform has a unique N‐terminal sequence of varying lengths. Longforms contain upstream conserved regions 1 (UCR1) and 2 (UCR2), shortforms only UCR2, and super‐short forms a truncated version of UCR2. Dead‐short forms consist of only a truncated and inactive catalytic domain.

**Fig. 3:**
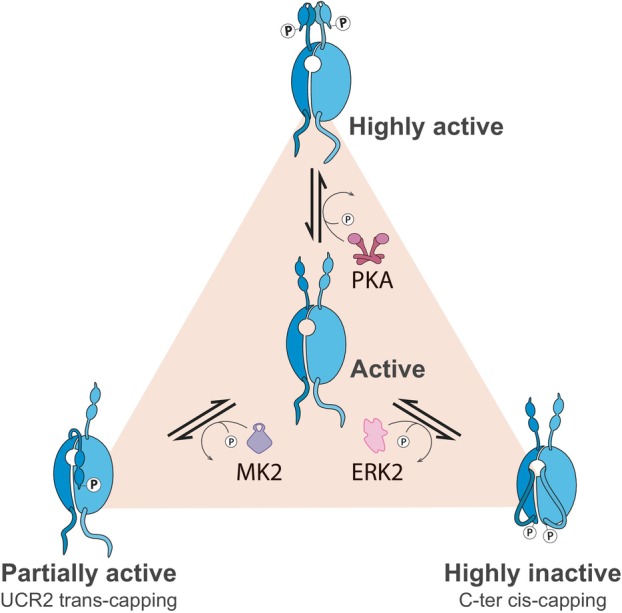
Regulation of PDE4 activity by phosphorylation. Protein kinase A (PKA)‐mediated phosphorylation in the Upstream Conserved Region 1 (UCR1) of long PDE4 isoforms modifies the electrostatic interactions between UCR1 and UCR2, leading to an increase in the cAMP hydrolytic activity of the enzyme. In addition to PKA, phosphorylation of UCR1 by AMPK, Cdk5, and SIK1 can similarly modify the stability of these N‐terminal domains and activate PDE4. MK2 action on PDE4 triggers a different conformational change that prevents binding of several PDE4 partners, such as PKA or DISC1. The reduction of PDE4 activity could be a consequence of the transcapping of the UCR2 regulatory helix from one monomer to the opposite active site in the dimer. The catalytic domain of PDE4s can also be blocked by the C‐terminal region in an intramolecular mode (cis‐capping), as these ends are too short to reach the monomer partner. This could be the mechanism responsible for the inhibitory effect of ERK2 phosphorylation at the end of the conserved PDE4 catalytic domain.

PDE4 isoforms can be separated into long, short, super‐short, and dead‐short enzymes depending on which regulatory domains are expressed (Fig. 2). Long PDE4 isoforms contain both upstream conserved region 1 (UCR1) and upstream conserved region 2 (UCR2) regions; all short forms lack UCR1, and super‐short isozymes also have truncated UCR2s, whereas dead‐short isoforms have a section of the catalytic site missing [3]. Additionally, each individual isoform has a unique N‐terminal sequence that may act as a ‘postcode’ to locate the enzyme to discrete intracellular locations. The presence of UCR1 dictates whether PDE4 dimerization can occur, with PDE4 long forms existing as dimers and all the shorter species as monomers [7]. Resolution of the crystal structure of the PDE4 dimer in 2010 [8] provided researchers with a scaffold around which to build appropriate mechanisms whereby individual PTMs may regulate the activity of the enzymes. Before this, the consequences of many PTMs were known, but the molecular mechanisms by which they were manifested could only be guessed at. In short, the structure of the PDE4 dimer showed there were two different ways in which PDE4 could be auto inhibited. First, the UCR1‐UCR2 module of one partner in the dimer can occlude the active site of the other and effectively inhibit catalytic activity by restricting access to cAMP in a process termed ‘trans‐capping’ [8]. Second, the C‐terminal lobe of PDE4 enzymes can block its own active site by ‘cis‐capping’ (Fig. 3). Both processes are differentially regulated by specific PTMs, which will be covered in the following sections.

## PDE4 phosphorylation

The first indication that PDE4 enzyme activity could be altered via phosphorylation was recorded when researchers noted a sharp increase in PDE4 activity following activation of the prostaglandin receptor [9]. Subsequent immunopurification of PDE enzymes from cell lysates allowed identification of PDE4D subfamily involvement. Further experimentation showed that the isoform PDE4D3 could be isolated, phosphorylated, and activated by co‐incubation with active protein kinase A (PKA). At that point, a conformational change following phosphorylation was postulated as the phosphorylation also changed sensitivity to PDE4 inhibitors that competed with cAMP at the active site. Other work by the same group validated the molecular mechanism by showing that the kinetics of the PKA phosphorylation were close to that of the activation of PDE4D3 [10]. Clever use of site‐directed mutagenesis identified two putative PKA phosphorylation sites of PDE4D3, serine 13 in the isoform‐specific N‐terminal region and Serine 54 in the UCR1 region, with only Serine 54 being linked to the activation, whereas Serine 13 was found to be involved in targeting of PDE4 activity to A‐kinase anchoring proteins [11]. Substitution of Serine 54 on PDE4D3 with negatively charged amino acids that mimic the phosphorylation was also found to activate the enzyme, verifying previous work [12]. Confirmation that all long‐form PDE4 isoforms had a similar activating PKA site in UCR1 followed [13] and this indicated a universal feedback mechanism where cellular cAMP increases following a Gs‐coupled receptor activation drives PKA activation (Fig. 3). Subsequent PKA phosphorylation of long‐form PDE4 enzymes then restored cAMP concentrations to basal levels. This action enables sensitivity to another round of receptor agonist challenge. In this light, activation of PDE4s is a viable therapeutic strategy in diseases where too much cAMP exists due to PDE4 downregulation or loss‐of‐function mutations in the catalytic site. Small molecule activators that bind to long‐form PDE4 UCR1 regions to phenocopy the PKA activation [14] are currently being tested for utility in autosomal‐dominant polycystic kidney disease where an excess of cAMP drives cyst formation [15] and prostate cancer where loss of long‐form PDE4s contributes to loss of the androgen receptor and an aggressive growth phenotype [16]. In the latter case, the isoform PDE4D7 has been reported to have a major role [17] and in addition to the activating PKA site in UCR1, PDE4D7 also has another PKA site in its unique N‐terminal region (Serine 42) that can be detected under basal cAMP conditions [18]. In contrast to the UCR1 site, this unique N‐terminal PKA motif is inhibitory when phosphorylated and presumably promotes cAMP signaling when cyclic nucleotide concentrations are low.

Phosphorylation of PDE4 enzymes is not limited to those actioned by PKA. PDE4A5, for instance, can be phosphorylated in UCR1 (Serine 147) by MAPK‐activated protein kinase 2 (MK2) [19]. Although this modification does not alter the intrinsic PDE activity of the enzyme, prior phosphorylation at this site can block any activation conferred by PKA phosphorylation in the conserved UCR1 PKA site (Fig. 3). Subtle conformational changes following MK2 phosphorylation of PDE4A5 also change its binding partner profile, conceptually, to allow changes in cAMP dynamics at a local level [20]. The same serine and another Serine 5 amino acids downstream in UCR1 are also both phosphorylated in PDE4D by salt‐inducible kinase 1 (SIK1), leading to its activation and subsequent cAMP decrease in pancreatic β‐cells [21]. This action reduced insulin secretion, suggesting that PDE4D inhibition may be a therapeutic route to lower blood glucose levels by having the opposite effect, and in doing so, may promote glucose tolerance under high‐fat diet conditions. Interestingly, the second serine in the SIK1 motif can also be phosphorylated in PDE4B1 by cyclin‐dependent kinase 5 (Cdk5) [22], and this too leads to an activation of PDE activity to enhance cAMP hydrolysis. When this PDE4 activation is reduced in the brain of mice by virally mediated knockout of Cdk5, mice exhibited antidepressive behaviors in stress tests such as forced swimming, tail suspension, and social defeat tests, suggesting a new therapeutic target for clinical conditions such as depression. Mechanistically, the activation of PDE4 by Cdk5 controls a pool of localized cAMP that influences the competence of neurotransmitter‐filled synaptic vesicles [23]. This suggests that presynaptic plasticity may be enhanced following PDE4 inhibition, and this may at least partially rescue behavioral deficits in animal models.

In the heart, it has been shown that calcium/calmodulin‐dependent protein kinase II (CaMKII) can phosphorylate and activate PDE4D to limit basal cAMP and counteract the increased amounts of cAMP produced after β‐adrenergic receptors are activated [24] in cardiac myocytes. The CaMKII phosphosite(s) on PDE4D are distinct from those utilized by PKA and are specific to PDE4D, as the effects of CaMKII inhibition on cAMP action were nullified in PDE4D knockout mice but still observed in PDE4B knockout mice. This novel CaMKII‐PDE4 axis was recognized as a novel point of crosstalk between the calcium and cAMP second messenger signaling systems, where CaMKII could be in the ascendency following depression of PKA signaling via PDE4D activation [24].

Another example of kinase‐mediated activation of PDE4 is the action of AMPK on PDE4B [25]. Small molecule AMPK activators were observed to reduce glucagon‐stimulated cAMP levels and attenuate PKA phosphorylation of downstream substrates. Analysis of the phosphorylation sites using mass spectrometry picked out three main sites for AMPK modification, 2 in UCR1 (with one being the same as the PKA site) and one at the start of the catalytic domain. All sites are conserved across species and in all subfamilies (PDE4A, B, C, D), suggesting that AMPK phosphorylation of PDE4s is ubiquitous. As PDE4B is expressed at a significantly higher degree in hepatocytes, more work on that subfamily should better pinpoint the role of this enzyme in metabolic diseases such as type 2 diabetes. Interestingly, PDE4B knockout mice exhibit a lean phenotype and lower TNF‐induced inflammation in white adipose tissue [26] but no difference in insulin sensitivity over wild‐type littermates.

Activation of PDE4D9 during mitosis via multisite phosphorylation combines the action of two kinases outlined above (AMPK, MK2) and ERK (see below) and an unknown kinase [27]. As cAMP and PKA activity are known to be important components in the coordination of cell cycle regulation [28], the spatial and temporal constraint of PDE4 activity during the individual phases is critical and a mechanism involving four different kinases could conceptually allow a complicated but measured fine control strategy directed by the formation of localized signaling complexes.

The unique range of possibilities for PDE4 enzyme regulation was dramatically increased following the discovery of an extracellular signal‐regulated MAP kinase (ERK) phosphomotif (PxS/TP) in the catalytic site of PDE4 enzymes [29]. Phosphorylation of PDE4D3 within this motif drastically reduced PDE4D activity, and this effect was abolished when the phosphoserine was substituted (Fig. 3). Additionally, the inhibition in activity conferred by ERK activation was transient, as the subsequent increase in cAMP activated PKA, triggering phosphorylation in UCR1, and promoting PDE4 activation to counteract the initial inhibition. Subsequently, two docking sites for ERK were found on the PDE4 catalytic unit on either side of the phosphomotif. A canonical kinase interaction motif (KIM) positioned on a surface facing β‐hairpin loop worked in cooperation with a FQF ERK specificity site located on an exposed alpha‐helix to efficiently position the kinase to modify the PDE4 [30]. The ERK consensus site could be found in only three of the four PDE4 subfamilies, with PDE4A isoforms lacking the ability to be phosphorylated [31]. It should be noted that despite the lack of phosphorylation of the native PDE4A, a recombinant fragment of PDE4A can be phosphorylated by ERK2, which could indicate the presence of other ERK sites in PDE4A, as this site is conserved in evolution [32]. ERK‐induced PDE4 inhibition was observed for all long isoforms (except PDE4A), with lesser inhibition noted for super‐short enzymes. Intriguingly, short isoforms were activated by ERK phosphorylation, providing different signaling outcomes in regions of cells that differentially express short or long‐form PDE4s. This concept was nicely illustrated when monocytes (which express mostly long PDE4s) differentiated into macrophages that predominantly express the short PDE4B2 isoform [33]. In the former cells, ERK activation depressed PDE4 activity, and the opposite was true following differentiation.

Finally, an unknown kinase acting downstream of phosphatidyl inositol 3‐kinase (PI3K) could phosphorylate PDE4D3 at the start of the catalytic unit (Serine 239), and this reprogrammed the expected PDE4 inhibition following ERK phosphorylation into an activation [34]. This process was shown to happen after exposure of cells to reactive oxygen species to allow activation of inflammatory responses.

## PDE4 ubiquitination and SUMOylation

In the last two decades, research into the modification of protein function via ubiquitin and ubiquitin‐like modifiers has become as prolific as work on kinase function. In this regard, PDE4 is known to be modified by both ubiquitin and SUMO (small ubiquitin‐like modifier).

Ubiquitination is a process where a protein is covalently but reversibly linked to a small 76 amino acid protein called ubiquitin via a cascade of enzymes including ubiquitin‐activating enzymes (E1), ubiquitin‐conjugating enzymes (E2), and ubiquitin‐protein ligases (E3) [35]. The latter class functions to couple the E2 enzymes and substrates for efficient ubiquitin transfer. Single or chains of ubiquitin can be added to lysine residues on targets to influence a range of protein functions. Often, the half‐life of a target is reduced as the ubiquitin tagged protein is degraded by the proteasome or in some cases the lysosome. Ubiquitination can also alter the binding partner profile of a substrate by altering affinities of protein–protein interactions. With respect to PDE4, it has been shown that this family belongs to a group of ‘extensively ubiquitinated proteins’ with multiple sites of modification identified [36]. Specifically, PDE4D half‐life is regulated by the ubiquitin proteasome system where degradation of the enzyme is directed by a cullin1‐containing E3 ligase complex. The recruitment is possible by the dual phosphorylation of the PDE by casein kinase 1 (CK1) and glycogen synthase kinase 3β (GSK3β) on a so‐called phosphodegron motif in the catalytic domain [37]. This degradation is opposed by PDE4D dephosphorylation facilitated by the calcium‐activated phosphatase calcineurin, and these complexes open new lines of cross talk between the calcium and cAMP signaling systems. Evidence also exists for PDE4B ubiquitination via action of the E3 ligase Smurf2 [38]. In this case, overexpression of the E3 ligase enhanced cAMP signaling via PDE4B degradation, and this in turn activated PKA to promote phosphorylation and activation of CREB and phospholamban. Although the exact ubiquitination sites were not determined, it was only the PDE4B subfamily that was affected, indicating the existence of a PDE4B‐specific binding site for Smurf2. These suggest that PDE4B may constitute a novel target for the treatment of liver fibrosis where cAMP is known to be protective [38].

**Fig. 4:**
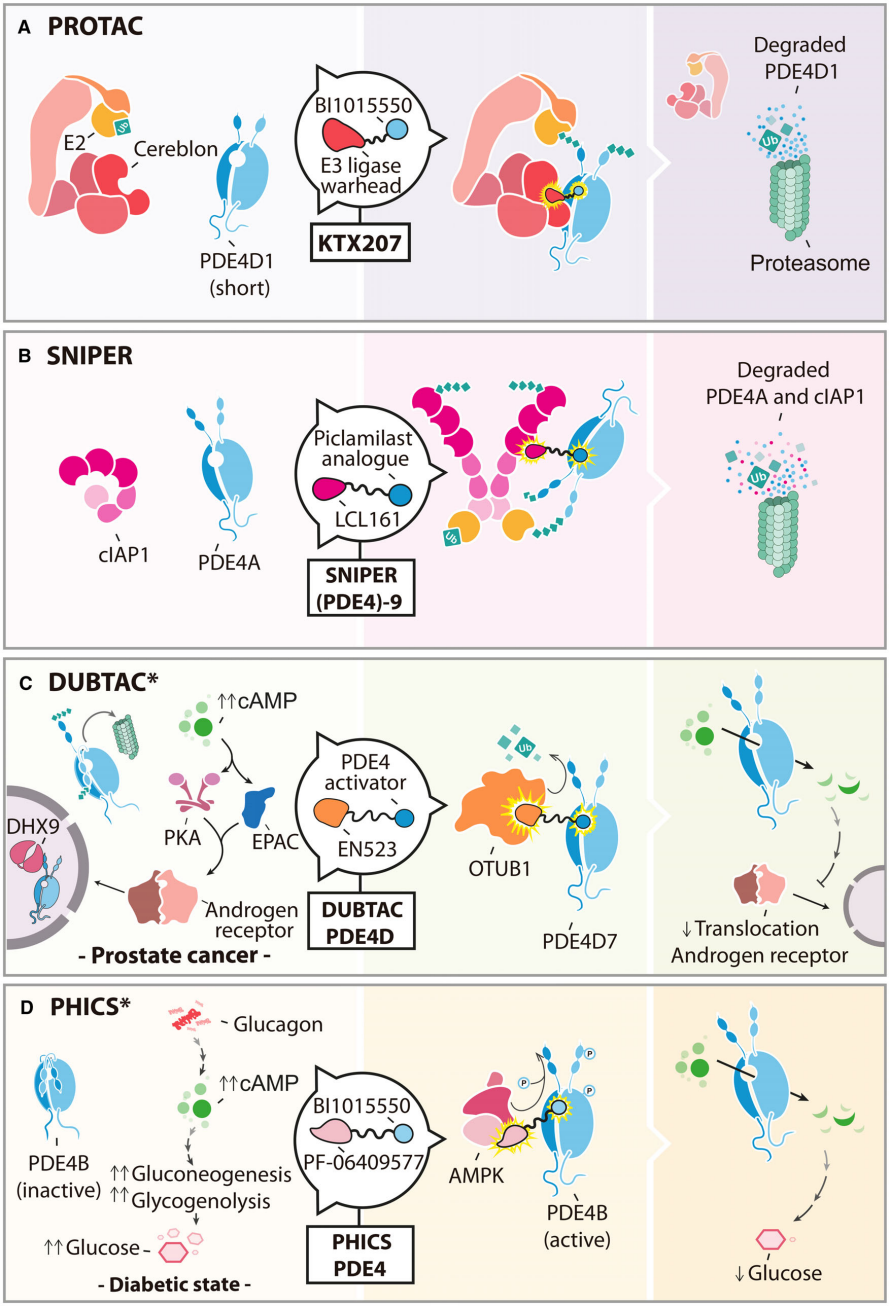
Strategies for targeting PDE4 PTMs using chemically induced proximity (CIPs). (A) KTX207 is a recently developed proteolysis‐targeting chimera (PROTAC) that evokes proteasomal degradation of PDE4, preferentially short forms. The chemical structure of this PROTAC consists of a cereblon‐E3 ligase warhead conjugated via a linker to BI 1015550, a PDE4 inhibitor with a high level of selectivity for PDE4B longforms and PDE4 shortforms. (B) Targeted ubiquitination of PDE4 has also been achieved using a specific and nongenetic IAP‐dependent protein eraser (SNIPER). SNIPER (PDE4)‐9 contains an LCL161 derivative that recruits cellular inhibitor of apoptosis protein 1 (cIAP1) linked to a piclamilast analogue. This captures PDE4 near the E3 ligase moiety, resulting in the degradation of both cIAP1 and PDE4. (C) Deubiquitinase‐targeting chimeras (DUBTACS) for stabilization of PDE4D7 represent a promising strategy in aggressive stages of prostate cancer. The progression of prostate cancer to an androgen‐unresponsive state is associated with reduced levels of PDE4D7 at the plasma membrane, which are a consequence of a downregulated gene expression combined with nuclear hijacking by DExH‐Box Helicase 9 (DHX9). PDE4D7 degradation could be halted by a DUBTAC incorporating a PDE4 activator, a linker region, and the ligand EN523 to recruit the deubiquitinase OTUB1. (D) The phosphorylation status of PDE4 could also be controlled using phosphorylation‐inducing chimeras small molecules (PHICS). These heterobifunctional molecules have been previously synthesized to force the proximity between the AMP‐activated protein kinase (AMPK) and a targeted substrate. Considering that AMPK antagonizes hepatic glucagon‐stimulated cAMP via activation of PDE4B, a PHICS including an AMPK allosteric activator (PF‐06409577 derivative) attached to BI 1015550 could be generated for the treatment of metabolic diseases such as type 2 diabetes. EPAC, exchange protein directly activated by cAMP; PKA, protein kinase A. Asterisks indicate CIP technology not yet developed.

The recent evolution of targeted protein degradation as a therapeutic strategy [39] has positioned ubiquitin‐directed PDE4 degradation as a viable route to develop a new range of modulators for the enzyme family. Proteolysis‐Targeting Chimera (PROTAC) recruit E3 ligases to a target protein to drive its destruction by the proteasome, and this has many advantages over conventional active site blockers that depend on occupancy (Fig. 4A). Early attempts to engineer a PROTAC for PDE4 used the E3 ligand attached to a PDE4 inhibitor as a ‘warhead’ [40] (Fig. 4B). The resulting active compound could reduce endogenously expressed PDE4A by approximately one‐half in cultured cells, and the catalytic action of the PROTAC was validated using a proteasome inhibitor. This work clearly showed that the concept of PDE4 PROTACs was viable, although this prototype lacked potency. More recent research has shown that the development of clinically used PDE4 inhibitors into PROTACs not only hugely increases the potency of the degrader versus the active site inhibitor but also allows advanced design, which can build in selectivity for different classes of PDE4, for example, short‐form vs long‐form specificity [41] (Fig. 4A). Another advantage of PDE4 PROTACs over conventional inhibitors is the longevity of action, as once inside the cell, the degrader sequentially eliminates PDE4 targets to ensure lack of rescue via transcriptional upregulation, as seen for drugs such as Roflumilast when used for chronic obstructive pulmonary disease (COPD) [42].

SUMOylation of a target on lysine residues is enabled by a similar cascade to that required for ubiquitination (E1, E2, and E3 ligase); however, there are distinct differences between the two processes [43]. SUMOylation of a protein usually does not promote its propensity to be degraded by the proteasome but can alter a cellular location, activity, or block ubiquitination. Additionally, SUMOylation often occurs on a defined motif, meaning that prediction of putative SUMO sites is easier when comparing these two sites of ubiquitination. In light of this, a single canonical SUMO site was discovered within the conserved catalytic sites of PDE4A and PDE4D [44]. Conjugation of SUMO to the PDE4s did not alter the intrinsic activity of the enzymes under resting conditions. However, it enhanced the PKA activation of long‐form PDE4A and PDE4Ds, presumably because the modification stabilized the open conformation of the dimer described above. Another effect of PDE4 SUMOylation was the protection against inhibition following ERK phosphorylation via the blockade of cis‐capping. In effect, a SUMOylated PDE4s can be considered as being in the fully active form until such time as the PTM is removed by deSUMOylating enzymes.

## PDE4 hydroxylation

Another PTM that has been shown to alter cAMP signaling via modification of PDE4 is hydroxylation [45]. Overexpression of a prolyl hydroxylase enzyme (PHD2) in cardiac myocytes increased cAMP by promoting a reduction in PDE4D protein expression and PDE4 activity. The opposite was true following PHD2 silencing. Conceptually, PDE4D hydroxylation, which was confirmed on multiple sites by mass spectrometry [45], would attract ubiquitin E3 ligases to send it for targeted degradation, as is the case for the HIF‐1 alpha protein [46]. Indeed, a proteasome inhibitor served to maintain PDE4D protein levels in PHD2 overexpressing cardiac myocytes. This evidence suggests that protein hydroxylation represents another avenue down which novel PDE4 inhibitors could be discovered.

## PDE4 irreversible cleavage by proteases

All PTMs discussed thus far have been reversible; however, one irreversible modification that is known to alter PDE4 function is proteolytic cleavage. Consensus sites for the protease Caspase 3 were identified within the unique N terminus of the PDE4A5 isoform, and it was shown that cleavage of the enzyme at Aspartate 72 happened during apoptosis [47]. Caspase 3 inhibitors prevented the PDE4 breakdown during apoptosis and retained the partial membrane localization of the enzyme, which was attenuated following protease action. As cAMP is known to be anti‐apoptotic in certain situations and pro‐apoptotic in others, the redistribution of a single PDE4 isoform following N‐terminal cleavage may be enough to increase a highly localized pool of the second messenger that enables apoptosis. Certainly, discretely positioned complexes containing cAMP effector proteins and PDE4 are known to influence apoptotic events [48] and the unhooking of membrane‐bound PDE4A5 by Caspase 3 during times of high cAMP may breach the activation threshold of such signalosomes.

## ‘Omics’ identification of PTMs involving PDE4 PTMs

Advances in various ‘omics’ techniques and improved accuracy of artificial intelligence (AI) tools for predicting PTM sites [49] in the last decade have greatly expanded our awareness of the diversity of possible PTMs that exist to alter protein function or to act as a metabolic reserve for nutrients. There are now hundreds of different modifications that can occur on at least 13 of the 20 known amino acids [50]. Some screens for these different PTMs have detected previously unknown modifications of PDE4, and although many of these are uncorroborated, with the functional consequences for cAMP signaling unknown, these reports may act as interesting starting points to discover new targeted therapeutic approaches toward PDE4 inhibition or activation. *O*‐GlcNAcylation is a dynamic event, which involves the addition and removal of O‐linked *N*‐acetylglucosamine to serine and threonine residues of targets. It is notoriously difficult to study because of low stoichiometry, but when perturbed, it has been implicated in aberrant cell signaling events that result in immune disease [51]. An early study, using enzymatic deglycosylation, had identified 474 *O*‐GlcNAcylated peptides that included a modified PDE4C enzyme sequence [52]. More recently, a more technologically advanced method, which was developed to enrich peptides modified by *O*‐GlcNAcylation, mapped 3293 events in 2470 proteins from HeLa cells [53]. Excitingly, three of these proteins were also identified as being enzymes from the PDE4B, 4C, and 4D subfamilies, and these data validated the original study. The *O*‐GlcNAcylated target sequences are in regions that are not completely conserved between subfamilies or are unique regions, suggesting that differential *O*‐GlcNAcylation could occur in a similar vein to SUMOylation, directing changes in only a subset of PDE4 isoforms. At this time, the functional consequences of PDE4 *O*‐GlcNAcylation are unknown; however, as the methods to investigate this PTM evolve further, its effect on cAMP signaling via PDE regulation will become clearer.

Another PTM that has attracted interest following advances in ‘click’ chemistry for fatty acids is acylation. Often added to lysine residues, covalent attachment of acyl groups can alter many aspects of a protein. In a study that looked at the differential acylation of proteins in the process of cell necroptosis, quantitative proteomics identified PDE4B peptides that were modified [54]. Interestingly, the sequence identified was in the unique N‐terminal region of PDE4B1, a long‐form PDE4B enzyme, which has recently been implicated in susceptibility to post‐traumatic stress disorder [55]. Modifications that affect only one isoform are rare, and this acylation event could be used to selectively target highly localized pools of PDE4B1 if technology advances.

## Conclusion and future perspective

The posttranslational modification of PDE4s is diverse and has been linked to the regulation of cAMP signaling in a variety of cell types and tissues via modulation of PDE4 activity and location. Although most is known about the phosphorylation of this enzyme family, more is starting to emerge about the functional consequences of more recently discovered modifications. Therapeutically, we already have compounds that mimic activating phosphorylations [14] and utilize the targeted degradation of PDE4s [40, 41] to create novel PDE4 activators (Fig. 4C) and negative modulators (Fig. 4A). Inhibitors that block the actions of enzymes, which catalyze PTMs have the disadvantage of targeting both disease‐related protein target modifications and nondisease targets concomitantly, and this can lead to side effects that restrict therapeutic utility. Modern approaches are now centered around targeting single modifications of disease‐causing proteins via chemical inducers of proximity (CIP) technology [56] (Fig. 2). Indeed, PROTACS (Fig. 4A) have been discussed here, but a cornucopia of similar technologies are being developed for nonubiquitin PTMs, for example, phosphorylation‐inducing chimeric small molecules [57] (PHICS, Fig. 4D), acetylations [58] (AceTAG), and *O*‐GlcNAcylations [59] (nanobody‐OGT) to name a few. Although these are in the early stage of development, the potential utility for PDE4 in the future may open a new dawn for targeted PDE4 inhibition/activation. The well‐studied phosphorylation patterns of PDE4 offer an attractive target for these new approaches, and PHICS have many features of PROTACS that may render them as apt choices to target this enzyme family. Specifically, the ability to design in specificity for PDE4 subfamilies, enable long‐lasting effects, and act with superior potency could mean that spatially restricted pools of PDE4 could be modified to alleviate disease‐causing, aberrant cAMP signaling [57]. For example, PHICS capable of recruiting AMPK have been developed [57], and this could be easily adapted for targeted modulation of PDE4 on disease dysregulated events where the PDE4 phosphorylation by this kinase plays an important role, like type 2 diabetes (Fig. 4D). In the opposite direction, phosphatases can be recruited by PhosTACs to dephosphorylate targets [60], and this may be useful in situations such as Acrodysostosis type II where over‐activated long‐form PDE4Ds depress cAMP and affect early development of patients [61]. Deubiquitinase‐Targeting chimeras (DUBTACs) [62] is another technology receiving much interest. Here, deubiquitinating enzymes are recruited to substrates to extend their half‐life by removing the ubiquitin, which targets them to the proteasome (Fig. 4C). This may be useful in disease situations where a deficiency in PDE4 enzyme activity leads to a disease‐causing, localized increase in cAMP such as prostate cancer [16], ovarian cancer [63] and polycystic kidney disease [64]. It should be noted, however, that DUBTACs are only effective on proteins that exhibit a relatively short turnover time, shaped by ubiquitination and the proteasome. To date, no literature has emerged that estimates the half‐life of different PDE4 subfamilies or isoforms, so we cannot be certain that this approach will be suitable for this enzyme family.

## Author contributions

MH, GST, and GSB were involved in the conception, design, and drafting of the manuscript. GST constructed and designed the figures. All authors approved the final version of the manuscript submitted for publication.

## Conflict of interest

The authors declare no conflict of interest.
